# Endophytic Actinobacteria Associated with* Dracaena cochinchinensis* Lour.: Isolation, Diversity, and Their Cytotoxic Activities

**DOI:** 10.1155/2017/1308563

**Published:** 2017-04-06

**Authors:** Nimaichand Salam, Thi-Nhan Khieu, Min-Jiao Liu, Thu-Trang Vu, Son Chu-Ky, Ngoc-Tung Quach, Quyet-Tien Phi, Manik Prabhu Narsing Rao, Angélique Fontana, Samira Sarter, Wen-Jun Li

**Affiliations:** ^1^State Key Laboratory of Biocontrol and Guangdong Provincial Key Laboratory of Plant Resources, School of Life Sciences, Sun Yat-Sen University, Guangzhou 510275, China; ^2^Department of Food Technology, School of Biotechnology and Food Technology, Hanoi University of Science and Technology, Hanoi, Vietnam; ^3^Yunnan Institute of Microbiology, Yunnan University, Kunming 650091, China; ^4^Laboratory of Fermentation Technology, Institute of Biotechnology, Vietnam Academy of Science and Technology, Hanoi, Vietnam; ^5^CIRAD, UMR QUALISUD, 34398 Montpellier, France

## Abstract

*Dracaena cochinchinensis* Lour. is an ethnomedicinally important plant used in traditional Chinese medicine known as dragon's blood. Excessive utilization of the plant for extraction of dragon's blood had resulted in the destruction of the important niche. During a study to provide a sustainable way of utilizing the resources, the endophytic Actinobacteria associated with the plant were explored for potential utilization of their medicinal properties. Three hundred and four endophytic Actinobacteria belonging to the genera* Streptomyces*,* Nocardiopsis*,* Brevibacterium*,* Microbacterium*,* Tsukamurella*,* Arthrobacter*,* Brachybacterium*,* Nocardia*,* Rhodococcus*,* Kocuria*,* Nocardioides*, and* Pseudonocardia* were isolated from different tissues of* D. cochinchinensis* Lour. Of these, 17 strains having antimicrobial and anthracyclines-producing activities were further selected for screening of antifungal and cytotoxic activities against two human cancer cell lines, MCF-7 and Hep G2. Ten of these selected endophytic Actinobacteria showed antifungal activities against at least one of the fungal pathogens, of which three strains exhibited cytotoxic activities with IC_50_-values ranging between 3 and 33 *μ*g·mL^−1^. Frequencies for the presence of biosynthetic genes, polyketide synthase- (PKS-) I, PKS-II, and nonribosomal peptide synthetase (NRPS) among these 17 selected bioactive Actinobacteria were 29.4%, 70.6%, and 23.5%, respectively. The results indicated that the medicinal plant* D. cochinchinensis* Lour. is a good niche of biologically important metabolites-producing Actinobacteria.

## 1. Introduction

Actinobacteria, especially the genus* Streptomyces*, are major producers of bioactive metabolites [1] and account for nearly 75% of the total antibiotic production available commercially [2, 3]. A few decades ago, antibiotics were considered as wonder drugs since they warded off deadly pathogens leading to eradication of infectious diseases. However, the unprecedented deployment of antibiotics over a period of time has resulted in evolution of multidrug-resistant pathogens. There is increasing attention to bioprospecting of Actinobacteria from different biotopes. With limiting bioresources, it is now imperative for search of unexplored or underexplored habitats. One such overlooked and promising niche is the inner tissues of plants, especially those with ethnomedicinal value [4–10].

The plant* Dracaena cochinchinensis* Lour. has been used as a traditional folk medicine in the oriental countries including China [11].* D. cochinchinensis* Lour. has many medicinally important properties, like antimicrobial, antiviral, antitumor, cytotoxic, analgesic, antioxidant, anti-inflammatory, haemostatic, antidiuretic, antiulcer, and wound healing activities [10, 12]. The plant is the source of deep red resin having medicinal properties which is also known as dragon's blood. The main components of dragon's blood are flavonoids and stilbenoids [13]. Apart from its medicinal use, it also finds applications as colouring materials and wood varnish [12]. The slow growth of the plant along with low yield of dragon's blood extracts, however, led to the destruction of large number of these plants, thereby endangering the plant. The current study described the diversity of culturable Actinobacteria associated with this medicinal plant and also indicated the cytotoxic potential of these Actinobacteria. The study, in a way, proposed a means for sustainable use of the plant resources without destroying the natural niche.

## 2. Materials and Methods

### 2.1. Sample Collection and Isolation of Endophytic Actinobacteria

Healthy plant samples (leaves, stems, and roots) of medicinal plant* D. cochinchinensis* Lour. were collected from four different provinces located in two countries: Pingxiang, Guangxi province, China (20°06′02′′N, 106°45′01′′E; elevation, 236 m); Xishuangbanna, Yunnan province, China (21°55′41′′N, 101°25′49′′E; 984 m); Bach Ma National Park, Thua Thien Hue province, Vietnam (16°9′55′′N, 107°55′19′′E; 1450 m), and Cuc Phuong National Park, Ninh Binh province, Vietnam (20°19′8′′N, 105°37′20′′E; 338 m). The plant samples were packed in sterile plastics, taken to the laboratory, and subjected to isolation procedures within 96 h. The samples were washed thoroughly with running tap water and in ultrasonic bath to remove any adhering soil particles and air-dried at ambient temperature for 48 h.

Two methods were employed for the isolation of the endophytic Actinobacteria using seven specific isolation media (Table 1).

**Method 1 .**

The plant parts of* D. cochinchinensis* Lour. were excised and subjected to a five-step surface-sterilization procedure: a 4 min wash in 5% NaOCl, followed by 10 min wash in 2.5% Na_2_S_2_O_3_, a 5 min wash in 75% ethanol, a wash in sterile water, and a final rinse in 10% NaHCO_3_ for 10 min. After drying thoroughly under sterile conditions, the surface sterilized tissues were disrupted aseptically in a commercial blender and distributed on isolation media [5, 7].

**Method 2 .**

The surface sterilized plant parts (1-2 g) were sliced, grounded with mortar and pestle, and mixed with 0.5 g CaCO_3_. The samples were kept in a laminar flow cabinet for 14 d, incubated at 80°C for 30 min, and plated onto isolation media [7].

Each medium was supplemented with nalidixic acid (25 mg·L^−1^), nystatin (50 mg·L^−1^), and K_2_Cr_2_O_7_ (50 mg·L^−1^) to inhibit the growth of Gram-negative bacteria and fungi; polyvinyl pyrrolidone (2%) and tannase (0.005%) were also added to improve the development of colonies on media. Colonies grown on these isolation media were selected and purified by repeated streaking on YIM 38 medium. The pure cultures were preserved as glycerol suspensions (20%, v/v) at −80°C and as lyophilized spore suspensions in skim milk (15%, w/v) at 4°C.

### 2.2. Identification and Diversity Profiling

For phylogenetic characterization, genomics DNAs of all isolates were extracted using an enzyme hydrolysis method. About 50 mg of the freshly grown culture was taken in an autoclaved 1.5 mL Eppendorf tube. To the culture, 480 *μ*L TE buffer (1x) and 20 *μ*L lysozyme solution (2 mg·mL^−1^) were added. The bacterial suspension was thoroughly mixed and incubated for 2 h under shaking conditions (160 rpm, 37°C). The mixture was treated with 50 *μ*L SDS solution (20%, w/v) and 5 *μ*L Proteinase K solution (20 *μ*g·mL^−1^) and kept on a water bath (55°C, 1 h). DNA was then extracted twice with phenol-chloroform-isoamyl alcohol (25 : 24 : 1 v/v/v), followed by precipitation with 80 *μ*L sodium acetate (3 mol·L^−1^, pH 4.8–5.2) and 800 *μ*L absolute ethanol. The resulting DNA precipitate was centrifuged at 4°C (12,000 rpm, 10 min), washed with 70% ethanol, and then air-dried. The extracted DNA was resuspended in 30 *μ*L TE buffer and stored at −20°C. PCR amplification for 16S rRNA gene from the extracted DNA samples was done using the primer pair PA-PB (PA: 5′-CAGAGTTTGATCCTGGCT-3′; PB: 5′-AGGAGGTGATCCAGCCGCA-3′) as described previously [14]. Amplified PCR products were purified and sequenced by Sangon Biotech (Shanghai). Identification of phylogenetic neighbours and calculation of pairwise 16S rRNA gene sequence similarities were achieved using the EzTaxon server (http://www.eztaxon.org/) [15] and BLAST analysis (http://blast.ncbi.nlm.nih.gov/Blast.cgi). The alignment of the sequences was done using CLUSTALW [16]. The phylogenetic tree was constructed using the aligned sequences by the neighbour-joining method [17] using Kimura 2-parameter distances [18] in the MEGA 6 software [19]. To determine the support of each clade, bootstrap analysis was performed with 1,000 replications [20].

### 2.3. Selection of Bioactive Actinobacteria Strains

Each of the isolated Actinobacteria was screened for antimicrobial activity and anthracyclines production. The antibacterial activities were evaluated against Methicillin-resistant* Staphylococcus epidermidis *(MRSE) ATCC 35984, Methicillin-resistant* Staphylococcus aureus *(MRSA) ATCC 25923, Methicillin-susceptible* Staphylococcus aureus *(MSSA) ATCC 29213,* Klebsiella pneumoniae *ATCC 13883,* Aeromonas hydrophila *ATCC 7966, and* Escherichia coli *ATCC 25922 using the agar well diffusion method [21]. Anthracycline productivity was screened using the pigment production test as described by Trease [22]. Based on the results of the two screenings, bioactive strains were selected for further assays.

### 2.4. Antifungal and Cytotoxicity Tests

Antifungal activity of the selected bioactive strains was tested against* Fusarium graminearum*,* Aspergillus carbonarius*, and* Aspergillus westerdijkiae* (strains producing the mycotoxins deoxynivalenol and ochratoxin A) [23, 24]. These test pathogens were provided by CIRAD, UMR QUALISUD, France, and maintained on Potato Dextrose Agar (PDA).

The cytotoxic activity of the selected strains was tested by sulforhodamine B (SRB) assay as described earlier [25–27]. The human breast adenocarcinoma (MCF-7) and human hepatocellular carcinoma (Hep G2) cells lines used for the test were procured from American Type Culture Collection (ATCC, Boulevard, Manassa, VA 20110, USA). Ellipticine was used as the positive control.

### 2.5. Screening for Biosynthetic Genes

Three sets of PCR primers A3F/A7R, K1F/M6R, and KS*α*F/KS*α*R were used for amplification of nonribosomal peptide synthetase (NRPS), polyketide synthase- (PKS-) I, and PKS-II specific domains [6, 28]. PCR amplifications were performed in a Biometra thermal cycler in a final volume of 25 *μ*L containing 0.2 *μ*mol·L^−1^ of each primer, 0.1 *μ*mol·L^−1^ of each of the four dNTPs (Takara, Japan), 2.5 *μ*L of extracted DNA, 0.5 unit of Taq DNA polymerase (with its recommended reaction buffer), and 10% of DMSO. Amplifications were performed according to the following profile: initial denaturation at 96°C for 5 min; 30 cycles of denaturation at 96°C for 1 min, primer annealing at either 57°C (for K1F/M6R, A3F/A7R) or 58°C (for KS*α*F/KS*α*R) for 1 min, and extension at 72°C for 1 min, followed by a final extension at 72°C for 5 min. The sizes of amplicons were 1,200–1,400 bp (K1F/M6R), 613 bp (KS*α*F/KS*α*R), and 700–800 bp (A3F/A7R).

## 3. Results

### 3.1. Isolation of Endophytic Actinobacteria

**Figure 1:**
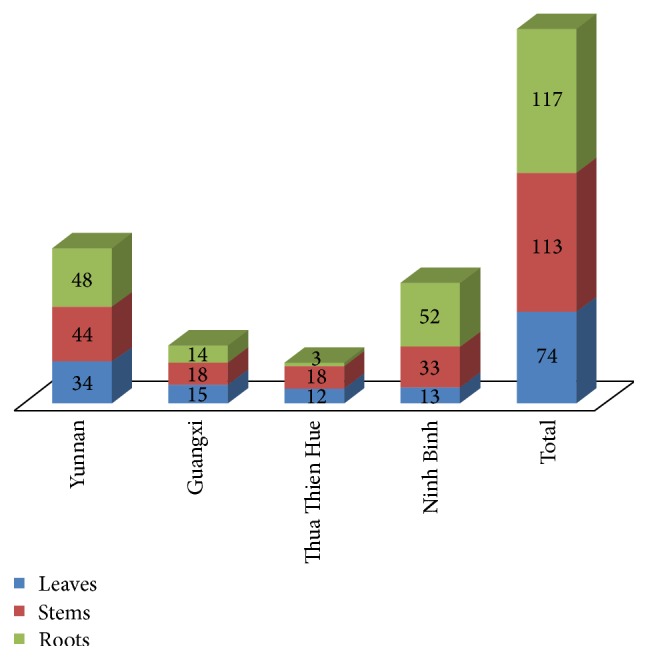
Distribution of endophytic Actinobacteria isolated from the different tissues of* Dracaena cochinchinensis* Lour. among the different sampling sites.

A total of 304 putative endophytic Actinobacteria were isolated from three different tissues of* D. cochinchinensis* Lour. The highest number of Actinobacteria was isolated from roots (117 strains, 38.49%), followed by stems (113 strains, 37.17%) and leaves (74 strains, 24.34%) (Figure 1). Among the sites, more Actinobacteria were isolated from Xishuangbanna (Yunnan province, China) and Cuc Phuong National Park (Ninh Binh province, Vietnam) (Figure 1).

**Figure 2:**
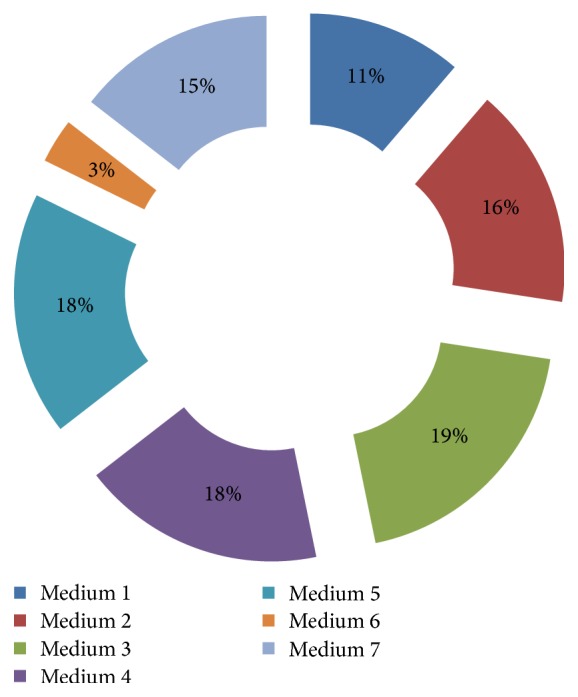
Effect of media on the isolation of endophytic Actinobacteria.

During the present study, Method 2 was found to be more suitable for the isolation of endophytic Actinobacteria from tissues of* D. cochinchinensis* Lour. and accounted for nearly 65% of the total isolation. All the media used in the current study, except for sodium propionate-asparagine-salt agar, were suitable for isolation of endophytic Actinobacteria (Figure 2).

### 3.2. Diversity Profiling

Based on the 16S rRNA gene sequence analysis, the most abundant Actinobacteria genera were* Streptomyces* (86.84%), followed by* Nocardiopsis* (4.93%),* Brevibacterium* (1.64%),* Microbacterium* (1.64%),* Tsukamurella* (1.64%),* Arthrobacter* (0.66%),* Brachybacterium *(0.66%),* Nocardia* (0.66%),* Rhodococcus* (0.66%),* Kocuria* (0.33%),* Nocardioides *(0.33%), and* Pseudonocardia *(0.33%). The relative abundance of the endophytic Actinobacteria among the different sites is shown in Table 2. Among the different sampling sites, Yunnan and Ninh Binh yielded the highest diversity, each contributing eight genera of Actinobacteria. Yunnan samples yielded the genera* Streptomyces*,* Nocardiopsis*,* Brevibacterium*,* Microbacterium*,* Brachybacterium, Rhodococcus*,* Kocuria*, and* Tsukamurella*, while Ninh Binh samples yielded* Streptomyces*,* Tsukamurella*,* Nocardiopsis*,* Arthrobacter*,* Nocardia*,* Brevibacterium*,* Nocardioides*, and* Pseudonocardia*. Thua Thien Hue samples contained* Streptomyces*,* Nocardiopsis*, and* Microbacterium*, while* Streptomyces *and* Nocardiopsis* were present in Guangxi samples.

### 3.3. Selection of Bioactive Actinobacteria Strains

All 304 Actinobacteria isolates were tested for antimicrobial activity and anthracycline production.  Table 3 represents the distribution of bioactive Actinobacteria. These bioactive strains were distributed in the genera* Streptomyces*,* Nocardiopsis*,* Nocardioides*,* Pseudonocardia*, and* Tsukamurella*. The genus* Streptomyces *possessed the highest proportion of isolates with antimicrobial activities. Anthracyclines are important group of antitumor antibiotics and are being used in cancer treatment [29, 30]. Of the 304 strains, 49 strains tested positive for anthracycline production.

**Figure 3:**
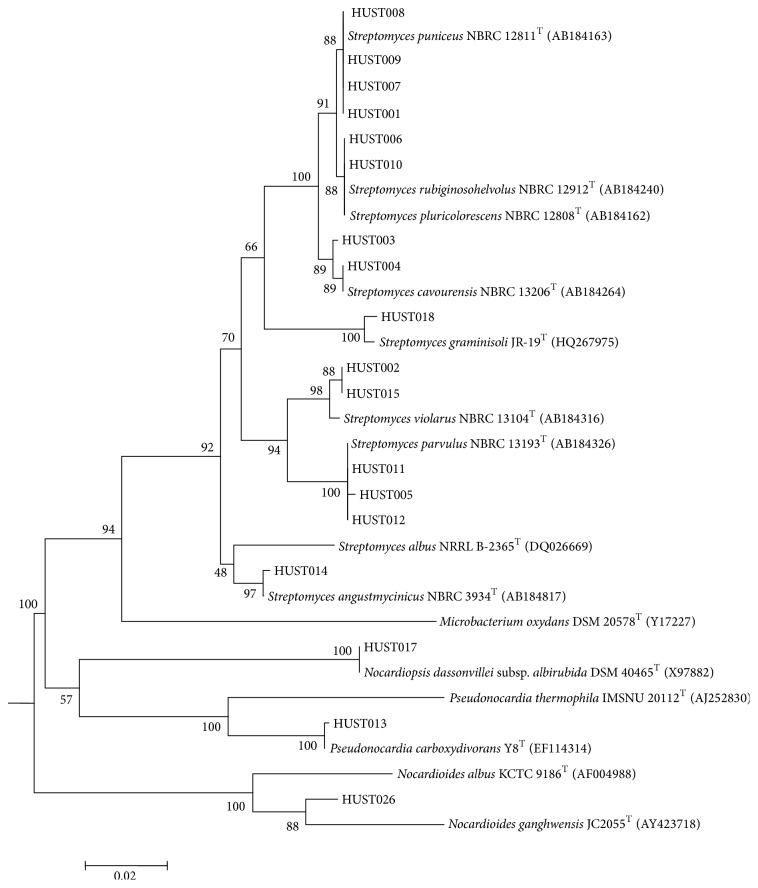
Neighbour-joining phylogenetic dendrogram based on 16S rRNA gene sequences showing the relationship of the selected 18 endophytic Actinobacteria with their closest species.

Based on the results of the bioactivity screening, 17 strains (HUST001-HUST011, HUST013-HUST015, HUST017, HUST018, and HUST026) were selected for further antifungal and cytotoxicity studies (Table 4). Of the 17 strains, 14 belonged to the genera* Streptomyces* while the rest comprised* Nocardioides*,* Nocardiopsis*, and* Pseudonocardia* (Figure 3).

### 3.4. Evaluation of Antifungal and Cytotoxicity Effects of the Bioactive Strains

Several strains among the selected bioactive Actinobacteria were positive for antifungal activities against the mycotoxins-producing* F. graminearum*,* A. carbonarius*, and* A. westerdijkiae* strains. Frequencies of the antifungal activities against the indicator fungal pathogens were as follows:* F. graminearum*: 58.8%;* A. carbonarius*: 41.2%; and* A. westerdijkiae*: 23.5%.  Table 5 summarizes the antifungal profile of the selected 17 strains.

Of the 17 strains, three strains (HUST001, HUST004, and HUST005) exhibited cytotoxic effects against the two tested human cancer cell lines, MCF-7 and Hep G2 (Table 5). Strain HUST004 showed significant inhibition toward MCF-7 cells with IC_50_-value of 3 *μ*g·mL^−1^, while strains HUST001 and HUST005 showed moderate activity with IC_50_-values of 19 and 25 *μ*g·mL^−1^, respectively. Against Hep G2 cell lines, IC_50_-values for the strains HUST004 and HUST005 were 10 and 33 *μ*g·mL^−1^, respectively. The remaining strains were inactive against the two cancer cell lines.

### 3.5. Screening of Biosynthetic Genes

All 17 bioactive strains were investigated for the presence of PKS-I, PKS-II, and NRPS genes. Frequencies of positive PCR amplification of the three biosynthetic systems were 29.41%, 70.59%, and 23.53%, respectively (Table 5). All these three genes were detected in two strains (HUST003, HUST004), which were identified as members of the genus* Streptomyces*. PKS-II gene was detected at highest frequencies in both* Streptomyces* and non-*Streptomycetes* genera, while PKS-I and NRPS genes were detected only in the genus* Streptomyces*.

## 4. Discussion

The plant source* D. cochinchinensis* is known for the production of dragon's blood [11]. Traditional practices of folk medicine involved extraction of dragon's blood from the plant. During its extraction, large scale exploitation of the plant is necessary owing to the low yield of plant's extract and slow growth of the plant, thereby resulting in destruction of large number of century old plant [13]. It is, therefore, imperative to search for alternative source of the plant's metabolites to preserve the plant in its natural niche. One such means is to study the endophytic microbes associated with the plant. In an earlier study by Cui et al. [35],* D. cochinchinensis* collected from Beijing, China, had been used to study the endophytic fungal diversity. The study resulted in the isolation of 49 fungal strains distributed into 18 genera. In another study of endophytic microbe associated with* D. cochinchinensis*, Khieu et al. [10] had isolated a* Streptomyces* strain, producing two potent cytotoxic compounds, from plant samples collected from Cuc Phuong National Park, Ninh Binh province, Vietnam. But neither of these studies described the diversity profile of the Actinobacteria communities living in association with the plant. As endophytic Actinobacteria from medicinal plants have been a major research area in the search of new antibiotic-producing strains [4, 7, 8, 36–39], we have selected the same plant source for in-depth analysis of Actinobacteria community structure. The present study resulted in the isolation of 304 Actinobacteria strains.

Many reports suggested that maximum endophytes were recovered from roots, followed by stems and leaves [9, 31–34]. Similar observation was found during our study whereby more number of isolates was obtained from roots than from stems or leaves (Table 6). This may be due to the fact that rhizospheric regions of the soil have higher concentration of nutrients. A report also suggested that microorganism enters various tissues of plant from rhizosphere and switched to endophytic lifestyles [40, 41]. Isolation of more isolates using the second method may be attributed to the enrichment of the samples with calcium carbonate. Qin et al. [7] have reported that calcium carbonate altered the pH to alkaline conditions which favour the growth of Actinobacteria.

Among various genera isolated,* Streptomyces *is predominantly present in the plant* D. cochinchinensis*. The finding is consistent with similar studies of endophytic bacteria [6, 9, 32, 33, 36]. In the present study, rare Actinobacteria of the genera* Arthrobacter*,* Brevibacterium*,* Kocuria, Microbacterium*,* Nocardia*,* Nocardioides*,* Nocardiopsis, Pseudonocardia*,* Rhodococcus*, and* Tsukamurella *were also isolated. Though* Arthrobacter*,* Brevibacterium*,* Microbacterium*,* Nocardia*,* Nocardioides*,* Nocardiopsis*,* Pseudonocardia*,* Rhodococcus*, and* Tsukamurella* have been reported as endophytic Actinobacteria of medicinal plant [6, 7, 31–34], this study forms the first report for the isolation of* Brachybacterium *and* Kocuria* (Table 6).

Endophytic Actinobacteria are often associated with antimicrobial properties [6, 7, 31]. This is shown by the high proportion of antibacterial activities by endophytic Actinobacteria associated with* D. cochinchinensis *Lour.: 23.03% against ATCC 35984, 23.26% against ATCC 25923, 25% against ATCC 29213, 23.68% against ATCC 13883, 32.43% against ATCC 7966, and 17.43% against ATCC 25922. Based on the preliminary bioactivity profile, a set of 17 Actinobacteria were further studied for antifungal and cytotoxic properties. Of the 17 strains selected, 10 strains were significant against* F. graminearum*, seven against* A. carbonarius*, and four against* A. westerdijkiae*. Similar findings have been reported in related studies of* Streptomyces *strains [42–44]. Four strains (HUST003, HUST004, HUST005, and HUST026) showed remarkable antifungal activity against all test fungi (Table 5). In contrast to above strains, HUST002, HUST006, HUST008, HUST009, HUST013, HUST015, and HUST017 did not show any antifungal activity.

In the study of Cui et al. [35], it was indicated that 71% of the fungal isolates obtained from* D. cochinchinensis* exhibited varied antitumor activities against five human cancer cell lines: HepG2, MCF7, SKVO3, Hl-60, and 293-T. Similarly, in the study of Khieu et al. [10], the compounds (*Z*)-tridec-7-3n3-1,2,13-tricarboxylic acid and Actinomycin-D produced by a* Streptomyces *sp. exhibited cytotoxic effect against two human cancer cell lines HepG2 and MCF-7. During the current study, three of the* Streptomyces* strains (HUST001, HUST004, and HUST005) produced potential cytotoxic activities. All the three studies on* D. cochinchinensis* indicated that the endophytic microbes associated with the plant are alternative sources for extraction of cytotoxic compounds. These studies further indicated that endophytic microbes can serve as a means for sustainable utilization of the plant resources by preserving the natural niche.

The cytotoxic abilities (IC_50_-values) of the three strains HUST001, HUST004, and HUST005 against the human cancer cell lines MCF-7 and/or Hep G2 range in between 3 and 33 *μ*g·mL^−1^. This finding is significant with reference to related studies [44–47]. Lu and Shen [45] isolated naphthomycin K from endophytic* Streptomyces *strain CS which exhibit cytotoxic activity against P388 and A-549 cell lines with IC_50_-values of 0.07 and 3.17 *μ*mol·L^−1^. Kim et al. [48] isolated salaceyins A and B from* Streptomyces laceyi* MS53 having IC_50_-values of 3.0 and 5.5 *μ*g·mL^−1^ against human breast cancer cell line SKBR3.

The biosynthetic genes are involved in microbial natural product biosynthesis. The antitumor drug bleomycin from* Streptomyces verticillus *ATCC 15003 involved a hybrid NRPS-PKS system [49]. Genomic analysis of the specific strain will, however, be necessary for illustration of the presence of biosynthetic gene clusters. Despite this fact, positive reaction for the amplification of specific domains for the three biosynthetic gene clusters is an indirect indication for the presence of the biosynthetic gene. In the present study, 13 of the 17 bioactive strains were found to have at least one of the three biosynthetic gene clusters. Among them, strains HUST003 and HUST004 showed positive results for the presence of PKS-I, PKS-II, and NRPS genes and also exhibited antifungal activity against all test pathogens (Table 5). Strains HUST006, HUST008, and HUST017 were negative both for the presence of PKS-I, PKS-II, and NRPS genes and for antifungal activity. The results indicated that the antifungal metabolites of these bioactive strains might be products of these biosynthetic genes. Li et al. [4] and Qin et al. [7] had reported that number of isolates having antimicrobial property need not correlate with the percentage of isolates showing the presence of PKS and NRPS gene and vice versa. Strains HUST002, HUST009, HUST013, and HUST015 did not show any antifungal activity but they encoded at least one of these biosynthetic genes. Similarly strain HUST014 was absent for PKS or NRPS gene products but showed antifungal activity.

## 5. Conclusions

Relatively fewer studies have been done to explore the endophytic microbes associated with medicinal plant. This study showed that endophytic Actinobacteria associated with the medicinal plant* D. cochinchinensis *Lour. could be an alternate source for production of bioactive compounds that were previously obtained from the medicinal plant. It thereby provides a sustainable way of utilizing the medicinal plant without destroying the plant.

## Figures and Tables

**Table 1:** Composition of the seven media used for the isolation of endophytic Actinobacteria from *Dracaena cochinchinensis* Lour.

Medium	Name and composition (g L^−1^of water)	Reference
1	*Tap water-yeast extract agar (TWYE)* Yeast extract 0.25, K_2_HPO_4_ 0.5, agar 15	[3, 5]

2	*Trehalose agar* Trehalose 6, KNO_3_ 0.5, CaCl_2_ 0.3, Na_2_HPO_4_ 0.3, MgSO_4_·7H_2_ O 0.2, agar 15	[5]

3	*Sodium propionate agar* Sodium propionate 2, NH_4_NO_3_ 0.1, KCl 0.1, MgSO_4_·7H_2_ O 0.05, FeSO_4_·7H_2_ O 0.05, agar 15	[5]

4	*Starch agar* Starch 2, KNO_3_ 1, NaCl 0.4, K_2_HPO_4_ 0.5, MgSO_4_·7H_2_ O 0.5, FeSO_4_·7H_2_ O 0.01, agar 15	[5]

5	*Citrate agar* Citric acid 0.12, ferric ammonium citrate 0.12, NaNO_3_ 1.5, K_2_ HPO_4_·3H_2_ O 0.4, MgSO_4_·7H_2_ O 0.1, CaCl_2_·H_2_O 0.05, EDTA 0.02, Na_2_CO_3_ 0.2, agar 15	This study

6	*Sodium propionate-asparagine-salt agar* Sodium propionate 4, asparagine 1, casein 2, K_2_HPO_4_ 1, MgSO_4_·7H_2_ O 0.1, FeSO_4_·7H_2_ O 0.01, NaCl 30, agar 15	[5]

7	*Dulcitol-proline agar* Dulcitol 2, proline 0.5, K_2_HPO_4_ 0.3, NaCl 0.3, MgSO_4_·7H_2_ O 1, CaCl_2_·2H_2_ O 1, agar 15	This study

**Table 2:** Distribution of endophytic Actinobacteria isolated from the different tissues of *D. cochinchinensis* Lour. among the different sampling sites.

Genera	Yunnan China	Guangxi China	Thua Thien Hue Vietnam	Ninh Binh Vietnam	Total
*Arthrobacter*	0	0	0	2	2
*Brachybacterium*	2	0	0	0	2
*Brevibacterium*	4	0	0	1	5
*Kocuria*	1	0	0	0	1
*Microbacterium*	4	0	1	0	5
*Nocardia*	0	0	0	2	2
*Nocardioides*	0	0	0	1	1
*Nocardiopsis*	8	1	2	4	15
*Pseudonocardia*	0	0	0	1	1
*Rhodococcus*	2	0	0	0	2
*Streptomyces*	104	46	30	82	262
*Tsukamurella*	1	0	0	5	6

*Total*	*126*	*47*	*33*	*98*	*304*

**Table 3:** Bioactivity profiles of the endophytic Actinobacteria isolated from *D. cochinchinensis* Lour.

Genera	Antimicrobial activity						Anthracycline production
	ATCC 35984	ATCC 25923	ATCC 29213	ATCC 13883	ATCC 7966	ATCC 25922	
*Arthrobacter*	0	0	0	0	0	0	0
*Brachybacterium*	0	0	0	0	0	0	0
*Brevibacterium*	0	0	0	0	0	0	0
*Kocuria*	0	0	0	0	0	0	0
*Microbacterium*	0	0	0	0	0	0	0
*Nocardia*	0	0	0	0	0	0	0
*Nocardioides*	0	0	1	1	0	0	1
*Nocardiopsis*	0	3	4	1	0	0	1
*Pseudonocardia*	0	0	1	0	1	0	1
*Rhodococcus*	0	0	0	0	0	0	0
*Streptomyces*	70	68	70	70	96	53	46
*Tsukamurella*	0	0	0	0	1	0	0

*Total*	*70*	*71*	*76*	*72*	*98*	*53*	*49*

*Proportion (%)*	*23.03*	*23.26*	*25.00*	*23.68*	*32.43*	*17.43*	*16.11*

*Note*. Number indicates number of isolates positive for the particular bioactivity.

ATCC 35984, Methicillin-resistant *Staphylococcus epidermidis *(MRSE); ATCC 25923, Methicillin-resistant *Staphylococcus aureus *(MRSA); ATCC 29213, Methicillin-susceptible* Staphylococcus aureus* (MSSA); ATCC 13883, *Klebsiella pneumoniae*; ATCC 7966, *Aeromonas hydrophila;* ATCC 25922, *Escherichia coli.*

**Table 4:** Isolation and characterization profile of the 17 selected endophytic Actinobacteria.

Strain	Sampling site^*∗*^	Isolation medium	Isolation method	Source	Accession number	Closest homologs	Pairwise similarity
HUST001	NB	3	2	Stem	KT033860	*Streptomyces puniceus* NBRC 12811^T^	100.0
HUST002	GX	2	1	Stem	KP317660	*Streptomyces violarus* NBRC 13104^T^	99.45
HUST003	TTH	5	1	Stem	KT033861	*Streptomyces cavourensis* NBRC 13026^T^	99.70
HUST004	YN	3	2	Root	KT033862	*Streptomyces cavourensis* NBRC 13026^T^	100.0
HUST005	NB	4	2	Stem	KT033863	*Streptomyces parvulus* NBRC 13193^T^	99.73
HUST006	NB	3	2	Stem	KT033864	*Streptomyces rubiginosohelvolus* NBRC 12912^T^	99.72
HUST007	YN	5	1	Root	KT033865	*Streptomyces puniceus* NBRC 12811^T^	100.0
HUST008	TTH	6	2	Stem	KT033866	*Streptomyces puniceus* NBRC 12811^T^	99.80
HUST009	YN	3	2	Stem	KT033867	*Streptomyces puniceus* NBRC 12811^T^	98.66
HUST010	YN	2	1	Root	KT033868	*Streptomyces pluricolorescens* NBRC 12808^T^	100.0
HUST011	GX	3	1	Root	KT033869	*Streptomyces parvulus* NBRC 12811^T^	100.0
HUST013	NB	4	1	Root	KT033870	*Pseudonocardia carboxidivorans* Y8^T^	100.0
HUST014	TTH	5	1	Root	KT033871	*Streptomyces augustmycinicus* NBRC 3934^T^	99.85
HUST015	TTH	7	2	Stem	KT033872	*Streptomyces violarus* NBRC 13104^T^	99.57
HUST017	YN	2	2	Leaf	KT033873	*Nocardiopsis dassonvillei *subsp. *albirubida *DSM 40465^T^	100.0
HUST018	NB	1	2	Root	KT033874	*Streptomyces graminisoli* JR-19^T^	99.45
HUST026	NB	1	2	Root	KT033859	*Nocardioides ganghwensis* JC2055^T^	98.26

^*∗*^YN, Xishuangbanna, Yunnan province, China; GX, Pingxiang, Guangxi province, China; TTH, Bach Ma National Park, Thua Thien Hue province, Vietnam; NB, Cuc Phuong National Park, Ninh Binh province, Vietnam.

**Table 5:** Antifungal, cytotoxic, and biosynthetic gene profiles of the 17 selected endophytic Actinobacteria isolated from *D. cochinchinensis* Lour.

Strain	Test pathogens			Cytotoxicity on MCF-7 (given in % inhibition)						Cytotoxicity on Hep G2 (given in % inhibition)						Biosynthetic genes		
	*Fusarium graminearum*	*Aspergillus carbonarius*	*Aspergillus westerdijkiae*	Concentration (*μ*g·ml^−1^)														
				10000	2000	400	80	16	IC_50_	10000	2000	400	80	16	IC_50_	PKS-I	PKS-II	NRPS
HUST001	+	−	−	101.74	94.48	70.47	63.70	48.84	19	103.92	103.29	102.75	56.25	12.42	68	−	+	−
HUST002	−	−	−	112.12	89.42	62.60	31.86	15.99	194	105.66	94.93	45.31	17.74	4.48	547	−	+	−
HUST003	+	+	+	106.71	97.45	67.50	44.38	19.33	120	109.97	109.29	90.54	54.56	−4.41	56	+	+	+
HUST004	+	+	+	105.40	88.68	73.52	62.87	56.94	3	109.18	107.70	103.23	87.50	62.08	10	+	+	+
HUST005	+	+	+	107.96	106.95	103.59	58.13	44.02	25	109.38	95.95	97.89	56.33	37.92	33	−	+	+
HUST006	−	−	−	78.54	17.16	5.59	−1.08	−10.50	5710	88.18	13.60	9.25	−2.98	−5.14	5745	−	−	−
HUST007	+	−	−	97.06	82.03	33.40	25.28	18.73	832	125.34	107.26	35.64	−3.78	−5.46	587	+	−	−
HUST008	−	−	−	99.71	81.92	42.76	30.32	11.34	399	105.41	103.72	27.53	8.78	−2.05	633	−	−	−
HUST009	−	−	−	94.51	84.24	26.54	16.43	8.34	870	121.62	104.90	18.83	−6.83	−17.04	688	+	+	−
HUST010	+	+	−	98.86	98.72	68.98	29.28	11.93	166	98.50	97.87	58.09	34.39	13.29	271	−	+	−
HUST011	+	+	−	98.10	54.71	47.71	39.48	24.42	695	85.25	47.37	27.53	13.06	−14.29	1721	+	+	−
HUST013	−	−	−	53.93	0.62	−3.91	−5.34	−6.81	9517	9.34	−3.28	−14.36	−19.50	−13.38	>10000	−	+	−
HUST014	+	−	−	91.67	80.05	52.38	43.63	11.27	249	109.90	106.17	86.86	46.85	−6.02	77	−	−	−
HUST015	−	−	−	99.05	83.68	70.60	37.20	15.59	129	101.49	97.87	76.25	32.02	−4.37	172	−	+	−
HUST017	−	−	−	22.66	−0.40	−1.00	−2.63	−8.75	>10000	−1.27	−1.70	−2.76	−1.54	−12.18	>10000	−	−	−
HUST018	+	+	−	42.02	6.29	4.41	−1.26	0.57	>10000	32.18	−0.88	−2.25	−12.44	−15.58	>10000	−	+	+
HUST026	+	+	+	92.08	86.55	37.72	28.64	22.61	623	104.63	86.23	38.05	21.95	10.72	691	−	+	−

**Table 6:** Comparative endophytic Actinobacteria diversity profile from different plant sources.

Plant sources	Number of isolates from different tissues				Diversity profile^*∗*^	Reference
	Leaves	Roots	Stems	Others		
*Artemisia annua *(Yunnan, China)	/	/	/	/	*Streptomyces* (123); *Promicromonospora* (26); *Pseudonocardia* (15); *Nocardia* (11); *Nonomuraea* (10); *Rhodococcus* (8); *Kribbella* (7); *Micromonospora* (7); *Actinomadura* (6); *Amycolatposis* (3); *Streptosporangium* (3); *Dactylosporangium* (2); *Blastococcus* (1); *Glycomyces* (1); *Gordonia* (1); *Kocuria* (1); *Microbispora* (1); *Micrococcus* (1); *Phytomonospora* (1)	[6]

*Maytenus austroyunnanensis *(Yunnan, China)	102	126	84	/	*Streptomyces* (208); *Pseudonocardia* (22); *Nocardiopsis* (21); *Micromonospora* (17); *Promicromonospora* (6); *Streptosporangium* (6); *Actinomadura* (4); *Amycolatopsis* (4); *Nonomuraea* (4); *Mycobacterium* (3); *Glycomyces* (2); *Gordonia* (2); *Microbacterium* (2); *Plantactinospora* (2); *Saccharopolyspora* (2); *Tsukamurella* (2); *Cellulosimicrobium* (1); *Janibacter* (1); *Jiangella* (1); *Nocardia* (1); *Polymorphospora* (1)	[9]

36 plant species (Chiang Mai, Thailand)	97	212	21	/	*Streptomyces* (277); *Microbispora* (14); *Nocardia* (8); *Micromonospora* (4); uncharacterized (27)	[31]

*Azadirachta indica *A. Juss. (Varanasi, India)	12	30	13	/	*Streptomyces* (27); *Streptosporangium* (8); *Microbispora* (6); *Streptoverticillium* (3); *Saccharomonospora* (3); *Nocardia* (2)	[32]

7 plant species (Mizoram, India)	6	22	9	2	*Streptomyces* (23); *Microbacterium* (9); *Leifsonia* (1); *Brevibacterium* (1); Uncharacterized (3)	[33]

26 species (Sichuan, China)	78	326	156	/	*Streptomyces, Micromonospora, Nonomuraea, Oerskovia, Promicromonospora, Rhodococcus*	[34]

*Dracaena cochinchinensis* Lour. (China and Vietnam)	74	117	113	/	*Streptomyces* (264); *Nocardiopsis* (15); *Brevibacterium* (5); *Microbacterium* (5); *Tsukamurella* (5); *Arthrobacter* (2); *Brachybacterium* (2); *Nocardia* (2); *Rhodococcus* (2); *Kocuria* (1); *Nocardioides* (1); *Pseudonocardia* (1)	This study

^*∗*^Number within parentheses indicates the number of strains from each genera; / indicates no data.

## References

[1] Bérdy J. (2012). Thoughts and facts about antibiotics: where we are now and where we are heading. *Journal of Antibiotics*.

[2] Rodrigues Sacramento D., Rodrigues Coelho R. R., Wigg M. D. (2004). Antimicrobial and antiviral activities of an actinomycete (*Streptomyces* sp.) isolated from a Brazilian tropical forest soil. *World Journal of Microbiology and Biotechnology*.

[3] Lee L.-H., Zainal N., Azman A.-S. (2014). Diversity and antimicrobial activities of actinobacteria isolated from tropical mangrove sediments in Malaysia. *The Scientific World Journal*.

[4] Li J., Zhao G.-Z., Chen H.-H. (2008). Antitumour and antimicrobial activities of endophytic streptomycetes from pharmaceutical plants in rainforest. *Letters in Applied Microbiology*.

[5] Li J., Zhao G.-Z., Qin S., Zhu W.-Y., Xu L.-H., Li W.-J. (2009). *Streptomyces sedi* sp. nov., isolated from surface-sterilized roots of *Sedum* sp. *International Journal of Systematic and Evolutionary Microbiology*.

[6] Li J., Zhao G.-Z., Huang H.-Y. (2012). Isolation and characterization of culturable endophytic actinobacteria associated with *Artemisia annua* L.. *Antonie van Leeuwenhoek*.

[7] Qin S., Li J., Chen H.-H. (2009). Isolation, diversity, and antimicrobial activity of rare actinobacteria from medicinal plants of tropical rain forests in Xishuangbanna, China. *Applied and Environmental Microbiology*.

[8] Qin S., Xing K., Jiang J.-H., Xu L.-H., Li W.-J. (2011). Biodiversity, bioactive natural products and biotechnological potential of plant-associated endophytic actinobacteria. *Applied Microbiology and Biotechnology*.

[9] Qin S., Chen H.-H., Zhao G.-Z. (2012). Abundant and diverse endophytic actinobacteria associated with medicinal plant *Maytenus austroyunnanensis* in Xishuangbanna tropical rainforest revealed by culture-dependent and culture-independent methods. *Environmental Microbiology Reports*.

[10] Khieu T.-N., Liu M.-J., Nimaichand S. (2015). Characterization and evaluation of antimicrobial and cytotoxic effects of *Streptomyces* sp. HUST012 isolated from medicinal plant *Dracaena cochinchinensis* Lour.. *Frontiers in Microbiology*.

[11] Wang X.-H., Zhang C., Yang L.-L., Gomes-Laranjo J. (2011). Production of dragon's blood in *Dracaena cochinchinensis* plants by inoculation of *Fusarium proliferatum*. *Plant Science*.

[12] Gupta D., Bleakley B., Gupta R. K. (2007). Dragon's blood: botany, chemistry and therapeutic uses. *Journal of Ethnopharmacology*.

[13] Fan L.-L., Tu P.-F., He J.-X., Chen H.-B., Cai S.-Q. (2008). Microscopical study of original plant of Chinese drug “Dragons Blood” *Dracaena cochinchinensis* and distribution and constituents detection of its resin. *Zhongguo Zhongyao Zazhi*.

[14] Li W.-J., Xu P., Schumann P. (2007). *Georgenia ruanii* sp. nov., a novel actinobacterium isolated from forest soil in Yunnan (China), and emended description of the genus *Georgenia*. *International Journal of Systematic and Evolutionary Microbiology*.

[15] Kim O.-S., Cho Y.-J., Lee K. (2012). Introducing EzTaxon-e: a prokaryotic 16s rRNA gene sequence database with phylotypes that represent uncultured species. *International Journal of Systematic and Evolutionary Microbiology*.

[16] Thompson J. D., Gibson T. J., Plewniak F., Jeanmougin F., Higgins D. G. (1997). The CLUSTAL X windows interface: flexible strategies for multiple sequence alignment aided by quality analysis tools. *Nucleic Acids Research*.

[17] Saitou N., Nei M. (1987). The neighbor-joining method: a new method for reconstructing phylogenetic trees. *Molecular Biology and Evolution*.

[18] Kimura M. (1983). *The Neutral Theory of Molecular Evolution*.

[19] Tamura K., Stecher G., Peterson D., Filipski A., Kumar S. (2013). MEGA6: molecular evolutionary genetics analysis version 6.0. *Molecular Biology and Evolution*.

[20] Felsenstein J. (1985). Confidence limits on phylogenies: an approach using the bootstrap. *Evolution*.

[21] Holder I. A., Boyce S. T. (1994). Agar well diffusion assay testing of bacterial susceptibility to various antimicrobials in concentrations non-toxic for human cells in culture. *Burns*.

[22] Trease G. E. (1996). *A Textbook of Pharmacognosy*.

[23] Boost M. V., O'Donoghue M. M., James A. (2008). Prevalence of *Staphylococcus aureus* carriage among dogs and their owners. *Epidemiology and Infection*.

[24] Khamna S., Yokota A., Lumyong S. (2009). Actinomycetes isolated from medicinal plant rhizosphere soils: diversity and screening of antifungal compounds, indole-3-acetic acid and siderophore production. *World Journal of Microbiology and Biotechnology*.

[25] Monks A., Scudiero D., Skehan P. (1991). Feasibility of a high-flux anticancer drug screen using a diverse panel of cultured human tumor cell lines. *Journal of the National Cancer Institute*.

[26] Shoemaker R. H., Scudiero D. A., Melillo G. (2002). Application of high-throughput, molecular-targeted screening to anticancer drug discovery. *Current Topics in Medicinal Chemistry*.

[27] Thao D. T., Phuong D. T., Hanh T. T. H. (2014). Two new neoclerodane diterpenoids from *Scutellaria barbata* D. Don growing in Vietnam. *Journal of Asian Natural Products Research*.

[28] Huffman J., Gerber R., Du L. (2010). Review recent advancements in the biosynthetic mechanisms for polyketide-derived mycotoxins. *Biopolymers*.

[29] Kremer L. C. M., Van Dalen E. C., Offringa M., Ottenkamp J., Voûte P. A. (2001). Anthracycline-induced clinical heart failure in a cohort of 607 children: long-term follow-up study. *Journal of Clinical Oncology*.

[30] Fischer C., Lipata F., Rohr J. (2003). The complete gene cluster of the antitumor agent gilvocarcin V and its implication for the biosynthesis of the gilvocarcins. *Journal of the American Chemical Society*.

[31] Taechowisan T., Peberdy J. F., Lumyong S. (2003). Isolation of endophytic actinomycetes from selected plants and their antifungal activity. *World Journal of Microbiology and Biotechnology*.

[32] Verma V. C., Gond S. K., Kumar A., Mishra A., Kharwar R. N., Gange A. C. (2009). Endophytic actinomycetes from *Azadirachta indica* A. Juss.: isolation, diversity, and anti-microbial activity. *Microbial Ecology*.

[33] Passari A. K., Mishra V. K., Saikia R., Gupta V. K., Singh B. P. (2015). Isolation, abundance and phylogenetic affiliation of endophytic actinomycetes associated with medicinal plants and screening for their in vitro antimicrobial biosynthetic potential. *Frontiers in Microbiology*.

[34] Zhao K., Penttinen P., Guan T. (2011). The diversity and anti-microbial activity of endophytic actinomycetes isolated from medicinal plants in Panxi Plateau, China. *Current Microbiology*.

[35] Cui J.-L., Guo S.-X., Dong H., Xiao P. (2011). Endophytic fungi from Dragon's blood specimens: isolation, identification, phylogenetic diversity and bioactivity. *Phytotherapy Research*.

[36] Cao L., Qiu Z., You J., Tan H., Zhou S. (2004). Isolation and characterization of endophytic Streptomyces strains from surface-sterilized tomato (*Lycopersicon esculentum*) roots. *Letters in Applied Microbiology*.

[37] Gu Q., Luo H., Zheng W., Liu Z., Huang Y. (2006). *Pseudonocardia oroxyli* sp. nov., a novel actinomycete isolated from surface-sterilized *Oroxylum indicum* root. *International Journal of Systematic and Evolutionary Microbiology*.

[38] Castillo U. F., Browne L., Strobel G. (2007). Biologically active endophytic streptomycetes from *Nothofagus* spp. and other plants in patagonia. *Microbial Ecology*.

[39] Duangmal K., Thamchaipenet A., Ara I., Matsumoto A., Takahashi Y. (2008). *Kineococcus gynurae* sp. nov., isolated from a Thai medicinal plant. *International Journal of Systematic and Evolutionary Microbiology*.

[40] Rosenblueth M., Martínez-Romero E. (2006). Bacterial endophytes and their interactions with hosts. *Molecular Plant-Microbe Interactions*.

[41] Compant S., Clément C., Sessitsch A. (2010). Plant growth-promoting bacteria in the rhizo- and endosphere of plants: their role, colonization, mechanisms involved and prospects for utilization. *Soil Biology and Biochemistry*.

[42] Rahman M. A., Islam M. Z., Khondkar P., Islam M. A. U. (2010). Characterization and antimicrobial activities of a polypeptide antibiotic isolated from a new strain of Streptomyces parvulus. *Bangladesh Pharmaceutical Journal*.

[43] Usha R., Ananthaselvi P., Venil C. K., Palaniswamy M. (2010). Antimicrobial and antiangiogenesis activity of *Streptomyces parvulus* KUAP106 from mangrove soil. *European Journal of Biological Sciences*.

[44] Jemimah Naine S., Subathra Devi C., Mohanasrinivasan V., Vaishnavi B. (2015). Antimicrobial, antioxidant and cytotoxic activity of marine *Streptomyces parvulus* VITJS11 crude extract. *Brazilian Archives of Biology and Technology*.

[45] Lu C., Shen Y. (2007). A novel ansamycin, naphthomycin K from *Streptomyces* sp.. *The Journal of Antibiotics*.

[46] Gontang E. A., Gaudêncio S. P., Fenical W., Jensen P. R. (2010). Sequence-based analysis of secondary-metabolite biosynthesis in marine actinobacteria. *Applied and Environmental Microbiology*.

[47] Rambabu V., Suba S., Manikandan P., Vijayakumar S. (2014). Cytotoxic and apoptotic nature of migrastatin, a secondary metabolite from *Streptomyces* evaluated on HepG2 cell line. *International Journal of Pharmacy and Pharmaceutical Sciences*.

[48] Kim N., Shin J. C., Kim W. (2006). Cytotoxic 6-alkylsalicylic acids from the endophytic *Streptomyces laceyi*. *Journal of Antibiotics*.

[49] Du L., Sánchez C., Chen M., Edwards D. J., Shen B. (2000). The biosynthetic gene cluster for the antitumor drug bleomycin from *Streptomyces verticillus* ATCC15003 supporting functional interactions between nonribosomal peptide synthetases and a polyketide synthase. *Chemistry and Biology*.

